# Degradation of Perfluorododecyl-Iodide Self-Assembled Monolayers upon Exposure to Ambient Light

**DOI:** 10.3390/nano14110982

**Published:** 2024-06-05

**Authors:** Lauren Colbeck Kirby, Jayant K. Lodha, Simon Astley, Dave Skelton, Silvia Armini, Andrew Evans, Anita Brady-Boyd

**Affiliations:** 1 Physics Department, Aberystwyth University, Aberystwyth SY23 3BZ, UK; 2 Semiconductor Technology and Systems, IMEC, Kapeldreef 75, B-3001 Leuven, Belgium

**Keywords:** self-assembled monolayer, area selective deposition, X-ray photoelectron spectroscopy, perfluorododecyl iodide, defluorination

## Abstract

Perfluorododecyl iodide (I-PFC12) is of interest for area-selective deposition (ASD) applications as it exhibits intriguing properties such as ultralow surface energy, the ability to modify silicon’s band gap, low surface friction, and suitability for micro-contact patterning. Traditional photolithography is struggling to reach the required critical dimensions. This study investigates the potential of using I-PFC12 as a way to produce contrast between the growth area and non-growth areas of a surface subsequent to extreme ultraviolet (EUV) exposure. Once exposed to EUV, the I-PFC12 molecule should degrade with the help of the photocatalytic substrate, allowing for the subsequent selective deposition of the hard mask. The stability of a vapor-deposited I-PFC12 self-assembled monolayer (SAM) was examined when exposed to ambient light for extended periods of time by using X-ray photoelectron spectroscopy (XPS). Two substrates, SiO_2_ and TiO_2_, are investigated to ascertain the suitability of using TiO_2_ as a photocatalytic active substrate. Following one month of exposure to light, the atomic concentrations showed a more substantial fluorine loss of 10.2% on the TiO_2_ in comparison to a 6.2% loss on the SiO_2_ substrate. This more pronounced defluorination seen on the TiO_2_ is attributed to its photocatalytic nature. Interestingly, different routes to degradation were observed for each substrate. Reference samples preserved in dark conditions with no light exposure for up to three months show little degradation on the SiO_2_ substrate, while no change is observed on the TiO_2_ substrate. The results reveal that the I-PFC12 SAM is an ideal candidate for resistless EUV lithography.

## 1. Introduction

Modern nanoelectronics relies on top–down patterning methods involving a repetitive sequence of deposition, photolithography, and etching steps [1,2,3]. However, the industry has recently been facing significant challenges in keeping up with Moore’s law [4,5] when using these conventional lithography techniques for patterning at critical dimensions. Traditional photolithography uses UV light to transfer patterns from a hard mask onto a photoresist covered-substrate, inducing a chemical change between the exposed and unexposed areas [6]. Hard masks are built from materials with high etch contrast relative to the underlying stack. Subsequently, the exposed substrate can undergo additional processes, such as etching or ion implantation, to create the final structure. With each advancement in technology, the intricacy and number of these procedures grow, leading to significant challenges in terms of patterning techniques. Patterning at scales smaller than 10 nm using these top–down techniques faces a number of difficulties, including edge placement errors, decreasing throughput, complexity, pattern collapse, and photoresist non-uniformity [7,8,9,10,11,12,13]. Due to these issues, there is a push towards using extreme UV photolithography. EUV refers to radiation at 13.5 nm (92 eV) [14]. These high-energy photons possess significantly more energy than those used in standard UV photolithography, allowing for finer resolution to reach the smaller critical dimensions [15]. The move to highly energetic EUV processes opens up the possibility for different chemical reactions to happen during exposure. As EUV sources have a lower power than previous sources, their flux is also lower. Traditional photoresists are not fully compatible with this process; therefore, alternative materials must be studied that have a higher EUV absorption cross-section [16]. Iodine readily absorbs EUV photons, so incorporating halogens such as this into photoresist materials can increase EUV absorption [17]. Kosto et al. found that a substation of one hydrogen atom on a 2-methylphenol (MPh) molecule for an iodine atom led to a 4.6-fold increase in the EUV photoabsorption cross-section [18].

At the same time, there is interest in developing and moving towards bottom–up deposition methods like area-selective deposition (ASD), removing the need for multiple photolithographic steps [19]. ASD enables material deposition on predefined patterns after altering the local surface chemistry [7,20,21]. Atomic layer deposition (ALD), generally utilized to deposit the material of interest, is a cyclic process that grows films through successive pulses of a metal precursor with a co-reactant, such as water, in a layer-by-layer manner [22]. Selectivity can be achieved via the passivation of certain areas of the surface through the use of self-assembled monolayers (SAMs) [23]. The SAMs preferentially adhere to one area or material of a patterned surface, called the non-growth area. They act as both a physical and chemical barrier to block any subsequent deposition on this area while still allowing growth on other areas or materials on the surface [7]. SAMs are compact organic monomolecular layers that spontaneously adsorb on a surface, showing large-scale ordering via Van der Waals force once deposited [24]. SAMs are comprised of a head group with a strong affinity for the substrate, a backbone chain, and a terminal functional group. SAMs bond to the surface via their head group, with common head group/substrate pairs including alkane-thiols on gold and other noble metals [25,26,27], silanes on silicon dioxide (and some metal oxides), and phosphonates on metal oxides [28]. SAMs also offer a diverse array of functionalities, for example, modifying the surface wettability, corrosion resistance [29], adhesion, friction [30], conduction [31], and biocompatibility. One of the many attributes of SAMs for ASD is that they can be easily patterned using soft lithography [32,33] and have been used in many applications, from arrays of single cells to open microfluidics [34,35,36].

**Figure 1:**
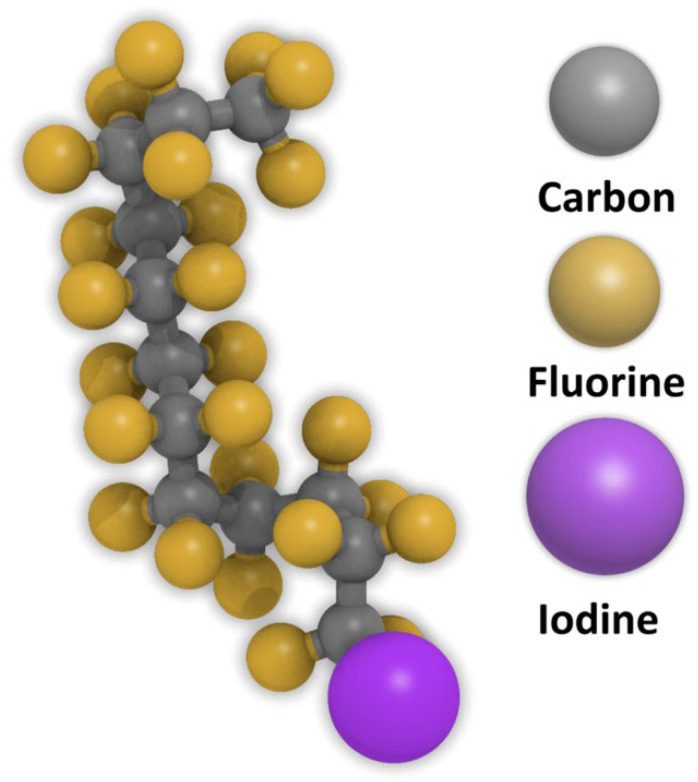
Chemical structure of the I-PFC12 molecule.

Recently, a novel method was introduced for the selective deposition of a hard mask layer within the growth region using ASD. This approach is employed to differentiate between the growth and non-growth regions following exposure of silane-based SAMs to EUV photolithography. To overcome the issues associated with photoresist, the study deposited these SAMs onto a TiO_2_ substrate, which is photocatalytic in nature. The photoactive surface aided the decomposition of the SAMs when exposed to EUV, thus producing a contrast in the exposed region [37]. Perfluorododecyl iodide (I-PFC12), as shown in Figure 1, is a SAM comprised of an iodine head group and a fluorocarbon backbone chain, making it a promising new candidate for this method. I-PFC12 exhibits intriguing properties, including ultralow surface energy due to a high fluorine content, the ability to modify silicon’s band gap, low surface friction, and suitability for micro-contact patterning [38]. As a result of the iodine atom, halogen bonding is expected to be the primary driver of the initial adsorption of I-PFC12, while dispersion forces play a key role in ensuring the long-term stability of the monolayers [38,39]. Halogen bonding is a non-covalent interaction between an electron-deficient halogen atom (often iodine) and a nucleophile or electron-rich species (often oxygen or nitrogen) [40,41,42,43].

This work investigates the stability of I-PFC12 SAMs on two different substrates, SiO_2_ and TiO_2_ when exposed to ambient light at different exposure times: from twenty-four hours up to one month. Even though the C-F bond is one of the strongest bonds in organic chemistry, environmental science studies show the degradation and defluorination of per and polyfluorinated chemicals in the presence of TiO_2_ and TiO_2_-based photocatalysts [44,45]. Testing the stability of I-PFC12 SAMs could help determine their suitability for ASD hard mask applications, removing the need for photoresist materials. Making use of the photocatalytic nature of TiO_2_, it is observed that the SAMs degrade via defluorination. Initially, the optimum vapor deposition parameters of the I-PFC12 were investigated using water contact angle (WCA) and spectroscopic ellipsometry (SE) to determine the hydrophobicity and film thickness of the SAM layer. Changes in the elemental composition of the I-PFC12 exposed to ambient light over time were investigated using X-ray photoelectron spectroscopy (XPS), along with any changes in the bonding environment. It was observed that over time and with exposure to ambient light conditions, fluorine decreases on both substrates, with a more pronounced decrease in the case of TiO_2_. Owing to the photocatalytic nature of TiO_2_, the SAMs degraded quicker on this substrate, making it an ideal choice for hard mask applications. Halogen bonding between the iodine head group and the OH-terminated substrate was investigated; however, there was no iodine observed for either substrate. Interestingly, when stored in a dark container with no ambient light, the I-PFC12 SAMs showed little signs of degradation. Although the iodine was not observed on either substrate in this study, the SAMs still degraded under ambient light, demonstrating a promising result on the use of I-PFC12 SAMs in EUV hard mask applications.

## 2. Materials and Methods

### 2.1. Substrate Preparation

In this study, 300 mm silicon wafers provided by SunEdison Semiconductors were used as the starting substrate for all samples. The Si substrates contained ~1.5 nm of a native oxide. A crystalline TiO_2_ layer measuring 7.5 nm in thickness was deposited via ALD using Titanium isopropoxide (Ti(OMe)_4_) as the precursor and water (H_2_O) as the co-reactant. This deposition process was conducted at a temperature of 300 °C. Further details of the substrates and TiO_2_ deposition process can be found in a previous study [37].

Both TiO_2_ and the SiO_2_ were UV ozone-cleaned in a Jelight UV ozone cleaner for 15 min prior to SAM deposition to remove any surface contaminants and to leave the surface rich in hydroxyl groups for the SAMs to adhere to. I-PFC12 SAMs were deposited on both TiO_2_ and SiO_2_ in a vapor phase in a dedicated Heratherm OM180 oven procured from Thermo Scientific with a vacuum pressure of 9–13 mbar.

Multiple depositions were performed to assess the optimum deposition time and temperature that yielded the I-PFC12 layer with the best quality. WCA and SE were used to characterize the surface hydrophobicity and thickness, respectively. To evaluate the deposition kinetics of the SAMs, WCA and thickness were measured for different deposition temperatures and times: in the range 100–150 °C and 1–2 h, respectively. The best film quality was deposited at a temperature of 120 °C and 100 °C for SiO_2_ and TiO_2_, respectively, for a deposition time of two hour on both.

### 2.2. SAM Characterization

A DataPhysics static water contact angle system was used to assess the hydrophobicity of the deposited SAM layer. The measurements were performed ex situ using de-ionized water, with a drop size of 2 µL and a dispensing speed of 1 µL/s. The WCA value was extracted from fitting using the SCA 20 software. UV ozone-cleaned TiO_2_ and SiO_2_ reference substrates were measured directly after pre-treatment. They showed a characteristic hydrophilic WCA value of <10°. The WCAs of the SAM-covered samples were compared to these reference samples. If an increase in hydrophobicity was observed, that was evidence of SAM deposition.

The thickness of the deposited SAM layer was measured ex situ using a J. A. Woollam RC2 Spectroscopic Ellipsometer system. The data were recorded at three incident angles, 65°, 70°, and 75°, with respect to the sample normal within a wavelength range of 200–2500 nm and an acquisition time of 5 s/angle. The beam divergence was 0.4° with a beam diameter of 3–4 nm. A model was fitted to the reference SiO_2_ and TiO_2_ substrates to find the thickness of the oxide layers. Once this was carried out, a Cauchy model was fit to determine the thickness of the SAM layers.

Following the deposition of I-PFC12 SAMs on the two different substrates, an investigation into their stability in ambient light was conducted using XPS. The samples were cleaved from larger three cm^2^ coupons into one cm^2^ coupons for ultrahigh vacuum XPS analysis. Once cleaved and before XPS characterization, the samples were stored in a dark container or were left for a certain length of time under ambient conditions. The base pressure of the XPS system was typically ~3 × 10^−9^ mbar. Measurements were recorded using an Al Kα (hν = 1486.6 eV) anode of a non-monochromatic PSP CTX400 flood gun X-ray source with a PSP HA50 energy analyzer at a pass energy of 20 eV for core-level scans and 90 eV for survey spectra. The angle of the X-ray source radiation and the analyzer were both 54° with respect to the sample normal. The peak fitting was performed using AAnalyzer peak-fitting software version 2.25. A Voigt peak, which is a combination of Gaussian and Lorentzian line shapes with a Shirley–Sherwood-type background, was used to fit the spectra [46]. The C 1s, F 1s, and O 1s peaks were fit with a Voigt singlet peak, and the Si 2p and Ti 2p were fit using Voigt doublet peaks. The spectra of the SiO_2_ substrate were referenced to the Si-Si peak at 99.1 eV. The spectra of the TiO_2_ substrate were referenced to the TiO_2_ peak at 458.8 eV, essentially using each underlying substrate for internal calibration.

XPS measurements were also scrutinized for any beam damage from the X-ray source. This was carried out by first recording the survey scans, followed by the core scans, and then a final set of survey scans. This allowed for approximately 2–2.5 h of X-ray exposure between the initial and final survey scans, allowing enough time for any damage to be observed. The atomic concentrations were then compared to the initial survey scans. After SAM deposition, the samples were placed into a dark container to ensure there was no exposure to ambient light. The samples were scanned as quickly as possible after SAM deposition. Then, three different samples were left in ambient light for different durations: 24 h, one week, and one month. Samples of I-PFC12 on both SiO_2_ and TiO_2_ were set aside and left sealed in the dark container and scanned after one and three months. Since the samples were cleaved from larger coupons, there were small variations observed in atomic concentrations due to regions of increased SAM density. Despite these variations, there is little impact on the overall trends of atomic concentrations across the surfaces.

## 3. Results

### 3.1. Optimal I-PFC12 SAM Deposition

**Figure 2:**
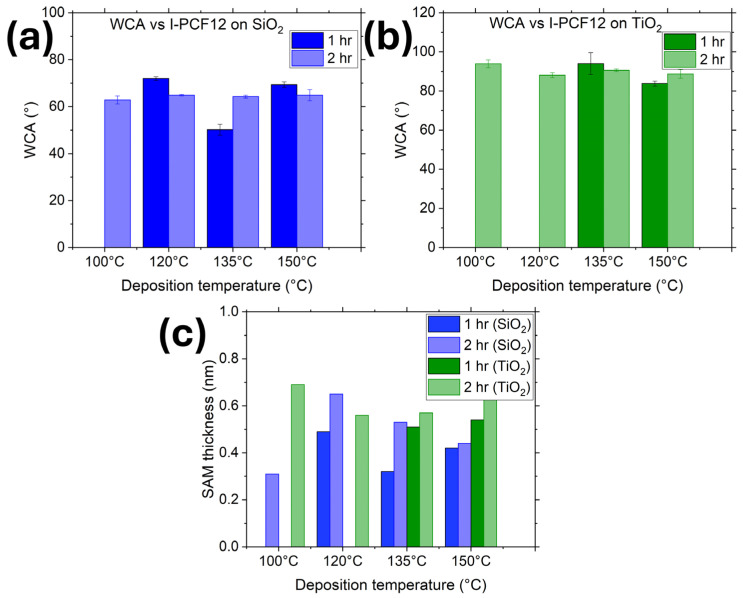
WCA of I-PFC12-derived SAMs on (**a**) SiO_2_ and (**b**) TiO_2_ substrates. (**c**) Thickness of SAMs deposited on SiO_2_ and TiO_2_ at temperatures ranging between 100 °C and 150 °C with deposition times of one and two hours.

I-PFC12-derived SAMs were deposited on both TiO_2_ and SiO_2_ surfaces via the vapor phase technique to be more compatible with current integration schemes used in the industry. For this, 100 mg of SAM was deposited at temperatures varying between 100 °C and 150 °C with deposition times of one and two hours (deposition for one hour at 100 °C is not included as an hour was not a sufficient length of time for the molecules to adsorb onto the surface at such a low temperature). Figure 2a,b illustrates the WCA of I-PFC12 deposited on SiO_2_ and TiO_2_ surfaces, respectively. A static contact angle test was performed to assess the hydrophobicity of the surface. It is notable that at a deposition time of one hour, the WCA varies considerably depending on the deposition temperature; however, at two hours of deposition, a consistent WCA is observed across all deposition temperatures. When examining the SAM thicknesses depicted in Figure 2c, it becomes apparent that the thickness is greater after the two-hour deposition period. The optimized conditions for SAM deposition on SiO_2_ involved a deposition temperature of 120 °C for two hours, resulting in a WCA of 64.9° ± 0.3° and a thickness of 0.65 nm. Conversely, for TiO_2_ surfaces, the optimized conditions were determined to be a deposition temperature of 100 °C for two hours, yielding a WCA of 93.9° ± 2° and a thickness of 0.69 nm.

### 3.2. I-PFC12 Degradation under X-rays

To assess what effect, if any, the X-rays induced on the I-PFC12 SAMs, samples from both substrates were exposed to X-rays for a period of time greater than two hours. Table S1 in the Supplemental Information (SI) displays the atomic concentrations for the I-PFC12 SAMs on the SiO_2_ and the TiO_2_ substrates. Five individual survey scans were recorded, followed by core-level scans. Five more survey scans were repeated, which amounted to approximately 2–2.5 h of X-ray exposure. The atomic concentrations of I-PFC12 on SiO_2_ showed a small decrease in the F 1s signal between survey one and survey five, reducing from 18.9% to 16.5%. Any subsequent X-ray exposure did not appreciably decrease the F 1s signal. After survey ten, the F 1s content was still 16.2%, showing that any decrease happens during the initial exposure. This points to the desorption of any unreacted I-PFC12 molecules from the deposition process that may be present on the surface [47]. Once these molecules have been removed, the I-PFC12 SAMs are stable, even after more than two hours of exposure to X-rays. Similarly, the SAM on TiO_2_ follows the same trend and shows a 2% decrease in F 1s, from 18.5% in the initial survey scan to 16.5% in the final survey scan. Again, this is attributed to the desorption of unreacted SAM molecules. As all small coupon-sized samples were cleaved from larger wafers, some variation was observed on a small number of samples. This could be due to an area where the SAM is less dense than other areas. However, the overall trends observed are the same, even if the atomic concentrations vary ever so slightly.

Interestingly, an I 3d peak was not observed on any sample or substrate at any time during the experiment, as shown in Figure S1 in the Supplementary Materials. To maximize the probability of observing the I 3d peak, it was scanned for first on all samples. However, it became clear that it was not present. Next, a large number of scans (50 scans) were recorded due to the low signal that would be expected from the I 3d. This did not yield the required results, and still, no iodine was observed. Single scans and a low number of scans (five scans) were performed in case the iodine was degrading fast due to X-ray exposure. However, again, no iodine was observed. It is unclear as to whether the iodine degrades under X-rays or if it diffuses upon exposure to air, or even a combination of both. Although there was no evidence of iodine, the monolayer remained on the surface. A previous study conducted by Keyun Shou et al. suggests that the monolayer forms due to halogen bonding between the iodine atoms in I-PFC12 and the oxygen atoms on the SiO_2_ surface. They postulate that dispersion forces help stabilize the monolayer on the surface. Although they did not have conclusive evidence of halogen bonding, they gave a detailed explanation as to why it is believed to be halogen bonding [38].

### 3.3. I-PFC12 Deposited on SiO_2_

Table 1 shows the atomic concentration taken from the survey spectra of the I-PFC12 deposited on the SiO_2_ when exposed to ambient light over time. A steady decrease in the F 1s from 19.1% to 12.9% is observed after one month of exposure to ambient light. This decrease in F 1s corresponds to an increase in C 1s intensity, with an initial concentration of 10.6% increasing to 14.2% after one month. A 3% increase in the O 1s is also observed during this time. This suggests that as the fluorine is being removed, there is an increase in the carbon signal from the fluorocarbon chain upon exposure to ambient light. Furthermore, the expected C:F ratio is 12:25, and in this work, it was found experimentally to be 10:19 on the SiO_2_ substrate, which is close to the expected value. The initial higher concentration of carbon indicates the presence of adventitious carbon upon atmospheric exposure. This ratio does change over time and with exposure to ambient conditions, increasing to 14:13 after a month of ambient light exposure. The change in ratio again points to the decomposition of the perfluorocarbon chain when exposed to ambient light.

**Figure 3:**
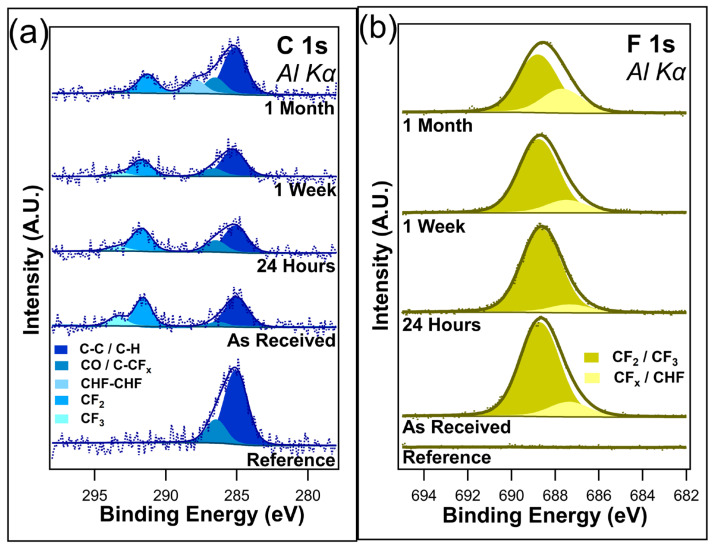
(**a**) C 1s and (**b**) F 1s of I-PFC12 on SiO_2_ display several different C-F bonds.

Figure 3a shows the unnormalized data for C 1s, and Figure 3b shows the F 1s core scans after different durations of ambient light exposure. C 1s contains four component peaks in the as-received sample, the twenty-four-hour sample, and the one-week sample: C-C/C-H at 284.8 eV, C-O/C-CF_x_ bonds at 286.2 eV, CF_2_ at 291.6 eV, and CF_3_ at 293.4 eV. All these peaks are consistent with those of the previous literature [48,49,50,51]. The peak at 286.2 eV was assigned to both C-O/C-CF_x_ bonds due to the sample being exposed to the atmosphere, meaning that the presence of C-O bonds cannot be discounted. After only twenty-four hours of ambient exposure, a decrease in the CF_3_ component peak was visible, corresponding to a very slight increase in the C-O/C-CF_x_ component peak. Very little change is observed between the twenty-four-hour exposure and the one-week exposure, with the C 1s spectra looking almost identical between the two samples. However, with one month of ambient exposure, the CF_3_ component peak disappeared from the spectrum, indicating the complete removal of the CF_3_ bonds. Interestingly, there is also the emergence of a CHF-CHF component peak at 287.9 eV. It is postulated that this peak emerges as the CF_x_ bonds in the fluorocarbon chain begin to break down. There is no evidence to suggest that I-PFC12 oxidizes over this time in either the C 1s or the O1s (Figure S2a in Supplementary Materials). The C 1s spectra show no evidence of the C-I bond between the head group of the SAM and the carbon chain.

The F 1s core-level spectra shown in Figure 3b contain two component peaks: CF_2_/CF_3_ at 688.7 eV and C-CF_x_/CHF at 687.3 eV [52]. The different CF bonds are not as distinguishable in the F 1s compared to the C 1s, as can be seen from the very broad F 1s envelope. As a result of this, the higher binding energy peak has been assigned to both CF_2_ and CF_3_ bonds. In contrast, the lower-binding-energy peak is thought to be a convolution of C-CF_x_ and CHF bonds. After only twenty-four hours of ambient exposure, a slight decrease in the intensity of the CF_2_/CF_3_ component peak was observed, mirroring the decrease seen in the C 1s of the CF_3_ component peak. After one week, a change in the ratio between the two component peaks starts to become visible as the CF_2_/CF_3_ component peak decreases. In the case of C 1s, a decrease in the CF_3_ component peak was observed. It is suggested that the CF_3_ bonds degrade due to the ambient conditions that leave C-CF_x_ bonds remaining. Following one month of ambient exposure, there is an obvious difference in the ratio between the two peaks. The higher-binding-energy peak has decreased in intensity while the lower-binding-energy peak has increased. This is further evidence of the degradation of the I-PFC12 SAMs, with the CF_3_/CF_2_ breaking down and forming C-CF_x_ and CHF bonds. An overlay of all the F 1s peaks is displayed in Figure S3 of the Supplementary Materials, where the decrease in F 1s intensity is clearly shown with increasing exposure time to ambient conditions.

The O 1s and Si 2p core-level scans are displayed in Figure S2 of the Supplementary Materials. The O 1s in Figure S2a predominantly consists of SiO_2_ at 532.4 eV, with evidence of some C-O bonds at 531.5 eV due to the presence of adventitious carbon. There does not appear to be any oxidation of the SAMs as no evidence was seen in the spectra for the formation of CO bonds or for FO bonds over the course of the experiment. The Si 2p core-level scans (Figure S2b) have two component peaks visible: Si bulk at 99.1 eV and SiO_2_ 103.1 eV. The Si 2p does not show any interaction with the SAMs, the iodine head group, or any carbon or fluorine bonds.

**Figure 4:**
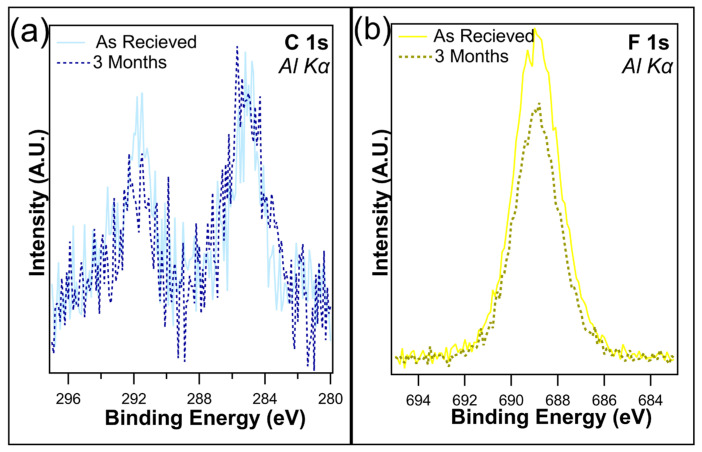
(**a**) C 1s and (**b**) F 1s of I-PFC12 on SiO_2_ as received and after 3 months of being kept in a dark container.

Several samples were maintained in dark containers and placed in a cabinet with no exposure to light for up to three months. The atomic concentrations (Table 1) reveal little change in the F 1s after one month when kept in dark conditions, with only a slight decrease from 19.1% to 18.6%, which is well within experimental error. This is consistent with the findings of Shou et al. [38]. Following three months in dark conditions, the loss of fluorine is just under 2%, as the F 1s concentration was recorded at 17.3%. Figure 4a,b overlays the unnormalized C 1s and F 1s, both as received and after 3 months of being kept in the dark. C 1s shows a slight decrease in the C-F bonds on the higher-binding-energy peak, which is consistent with the loss of CF_2_/CF_3_ bonds. A decrease in F 1s intensity was observed, corresponding to the decrease in C 1s. Overall, no change in peak shape was observed, demonstrating that although the CF_x_ bonds were being removed, there is no creation of any new chemical states.

### 3.4. I-PFC12 Deposited on TiO_2_

Table 2 shows the atomic concentration taken from the survey spectra of the I-PFC12 deposited on the TiO_2_ when exposed to ambient light over time. A 10.2% decrease in the F 1s from 17.6% to 7.4% is observed after one month of exposure to ambient light. The C 1s increases over time from 11.56% to 14.4%. A 5% increase in the O 1s was also observed during this time, while the Ti 2p remained fairly constant. This suggests that the SAMs degrade with ambient exposure due to the removal of the fluorine atoms, which reveals more of the TiO_2_ substrate. As previously stated, the expected C:F ratio is 12:25, and on the TiO_2_ substrate, it was found experimentally to be 12:17. This indicates that more carbon is present on the sample, pointing to the presence of adventitious carbon upon atmospheric exposure, which is similar to the case of the SiO_2_ substrate. This ratio does change over time and with exposure to ambient conditions, increasing to 10:7 after a month of ambient light exposure. The change in ratio again points to the decomposition of the perfluorocarbon chain when exposed to ambient light. The change in F content is more substantial on the TiO_2_ substrate than that observed on the SiO_2_ substrate, with a loss of 10.2% of the F 1s on TiO_2_ compared to a loss of 6.2% on the SiO_2_ substrate. It is proposed that this sharper decrease in F 1s is due to the photocatalytic nature of TiO_2_.

**Figure 5:**
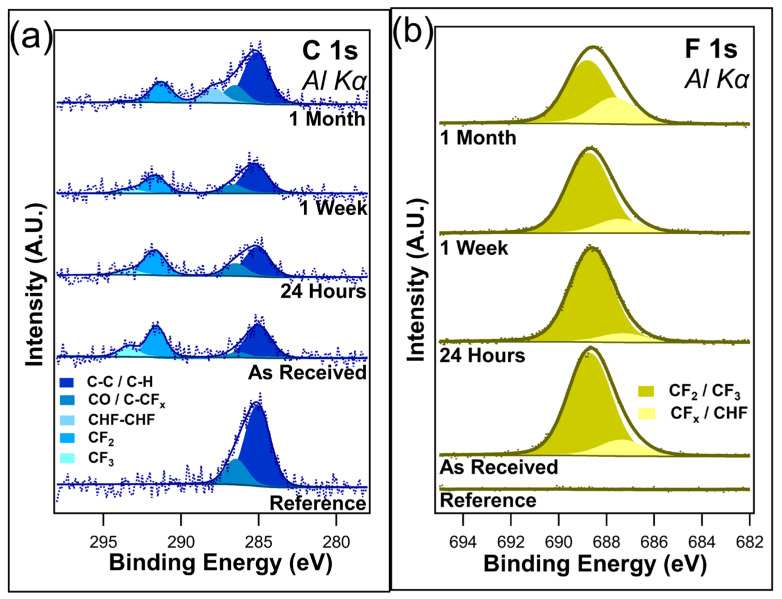
(**a**) C 1s and (**b**) F 1s of the I-PFC12 SAM deposited on a TiO_2_ substrate.

Figure 5a displays the C 1s, and Figure 5b displays the F 1s for the I-PFC12 SAMs on TiO_2_. Four component peaks were identified in the C 1s and were attributed to C-C/C-H at 284.8 eV, C-O/C-CF_x_ at 286.2 eV, CF at 288.9 eV, and CF_2_ at 291.6 eV binding energies [48,49,50]. The two lower-binding-energy peaks and the CF_2_ component peak are consistent with the SAMs on SiO_2_. However, no CF_3_ component peak was detected for any I-PFC12 SAM on the TiO_2_ sample that was characterized. Interestingly, a CF peak at a binding energy of 288.9 eV was observed for all samples [44,45].

Little difference was observed in the C 1s spectra between the as-received and the samples exposed to twenty-four hours of ambient conditions. Only a slight increase was observed in CF and C-O/C-CF_x_ peaks after one week. After one month, a decrease in the CF_2_ peak was observed, and the CF peak underwent a subtle increase. A change in intensity was also observed for the C-O/C-CF_x_ peak. There was possible evidence of a C=C peak emerging on the lower-binding-energy side after one month of exposure. Due to the high signal-to-noise ratio, this peak has not been included in the peak fitting but could give valuable insights into how the I-PFC12 SAMs degrade. It is thought that the emergence of this peak is evidence of an intermediate stage in the SAM defluorination process; this will be explained in more detail in the discussion section. The C 1s showed no evidence of a C-I bond between the head group of the SAM and the carbon chain.

The as-received F 1s core scan contains two component peaks: CF_2_ at 688.7 eV and CF/C-CF_x_ at 687.9 eV. Mirroring C 1s, there was little difference detected between the as-received and twenty-four-hour exposed samples. However, after one week, a small noticeable change occurred in the ratio of the two component peaks. The intensity of the CF/C-CF_x_ appeared to increase with respect to the CF_2_, reflecting what was observed in the C 1s spectra. After one month, the F 1s peak depreciated considerably, with the degradation of the CF_2_ component peak. Once again, this remains consistent with the trends observed in the case of C 1s. Figure S4a shows O 1s, and Figure S4b shows Ti 2p. These spectra remained constant for the entirety of the experiment, and no changes were observed.

**Figure 6:**
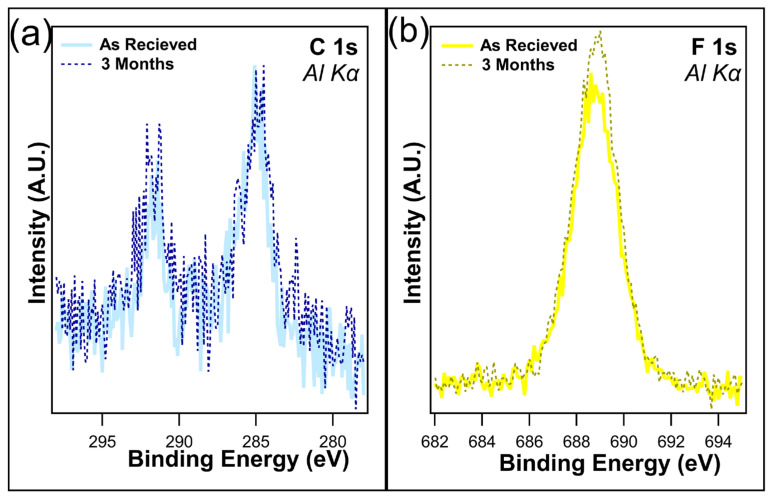
Overlays of (**a**) C 1s and (**b**) F 1s following one month and three months in dark conditions. No degradation of the SAMs was observed at this time.

From Table 2, a very small increase in both the C 1s and F 1s can be seen in the case of I-PFC12 on TiO_2_ after three months in dark conditions. Due to the nature of XPS, this 0.2% increase in carbon and 0.3% in fluorine is well within experimental error and was interpreted as no observable change or degradation in the SAM. The overlays of the C 1s and F 1s are displayed in Figure 6a and b, respectively; they compare the as-received samples and those that were kept for three months in dark conditions. An increase in intensity for both peaks was observed after three months, again demonstrating no signs of defluorination of the SAMs. Therefore, the SAMs are stable on TiO_2_ for up to three months in the dark, confirming that the changes observed are due to the ambient conditions and not just the SAMs degrading over time.

## 4. Discussion

The more pronounced defluorination of the I-PFC12 SAM on TiO_2_ compared to the SiO_2_ can be attributed to the photocatalytic nature of the TiO_2_ substrate. This is a well-known phenomenon and property of TiO_2_ that enables its many applications, from water splitting to use in solar cells; it is utilized here to actively degrade SAMs. A review by Schneider et al. provides a comprehensive overview of the mechanisms behind the photocatalytic nature of TiO_2_ and some of its practical uses [52]. Environmental science studies can provide valuable insights into the degradation of per- and polyfluorinated molecules. These molecules are heavy pollutants found in water and can bioaccumulate in both animals and humans. Considerable research has been carried out in recent years into how best to degrade and decompose these molecules. As they are similar in structure and composition to the I-PFC12 SAMs, they can be used as an analogy as to how the SAMs degrade. Bentel et al. looked at the defluorination of 34 differently terminated per- and polyfluorinated molecules and found differences in the decay of these molecules based on termination and chain length [53]. In particular, several studies have looked at using photocatalytic solutions to disassociate the C-F bonds found in these molecules. Of these, TiO_2_-based photocatalysts have been shown to be extremely effective [44,45].

Yamijala et al. [54] investigated the degradation of per- and polyfluoroalkyl substances using molecular dynamic simulations. Their findings demonstrate that excess electrons are the key to the defluorination process. These excess electrons can originate from oxygen vacancies in the TiO_2_ and from photoexcitation. Due to the wide band gap of TiO_2_, the charge carrier lifetime increases as the electron–hole recombination rate decreases. This gives a greater chance of electrons reaching the surface compared to other materials, including SiO_2_, which is known to be a poor photocatalyst. The results observed here demonstrate the superior photocatalytic behavior of the TiO_2_ substrates over the SiO_2_ substrate, as after one month of ambient light exposure, the SAMs decreased by 10.2% compared to 6.2%, respectively.

Figure 3 and Figure 5 reveal different pathways to degradation in relation to the I-PFC12 SAMs depending on the substrate they are deposited on. On the SiO_2_ substrate, the growth of an intermediate CHF-CHF bond is observed as the CF_3_ and CF_2_ peaks decrease. However, the emergence of a CF peak was observed on the TiO_2_ substrate, and there was an increase in the C-CF_x_ component peak. Despite the fact that the SiO_2_ substrate is an inefficient photocatalyst, it can absorb shortwave UV light. Following the UV ozone pre-treatment step, the surface is OH terminated. The SAM cannot bond to every available OH site due to the steric hindrance of the SAM molecules, and so some OH groups remain on the surface. The creation of electron–hole pairs due to light exposure allows for the creation of OH radicals as there are already OH groups present as well as absorbed water [55]. The created OH radicals are then free to interact with and degrade the SAM chain. It is postulated that these radicals can disassociate a C-F bond within the CF_2_ chain, leaving CHF in its place.

On the other hand, the TiO_2_ substrate has excess electrons, which can disassociate a C-F bond, as shown by Yamijama et al. Their simulations show that the dissociation of the C-F bond forms a C=C bond in the chain. Liu et al. [56]. also established that C-F bonds in the presence of a C=C bond can degrade much more rapidly than a C-F bond in the presence of a C-C bond. The more C=C bonds formed, the quicker the defluorination of I-PFC12 will happen. Although the signal to noise for the sample exposed to one month of ambient conditions is poor, the formation of a C=C cannot be ruled out. Figure S5 displays an alternate peak fitting for the C 1s after one month of ambient exposure in the case of TiO_2_. A component peak corresponding to C=C can be added on to the lower-bonding-energy side of the C-C/C-H peak. While a more conservative approach has been taken for peak fitting to keep it consistent with the other experimental steps, the emergence of a C=C peak cannot be fully ruled out. The inclusion of the C=C would experimentally confirm the previous studies of Yamijama et al. [54].

## 5. Conclusions

In conclusion, the stability of I-PFC12 SAMs deposited on both SiO_2_ and TiO_2_ was investigated when exposed to ambient light conditions for different durations of up to one month. No obvious X-ray damage of the SAM molecules was detected following 2–2.5 h of X-ray exposure. The iodine head group was not observed on any sample, whether deposited on SiO_2_ or TiO_2_, with no evidence of the C-I bond between the head group of the SAM and the carbon chain observed in the spectra. This indicates that any degradation of the SAMs is not due to iodine’s readiness to absorb EUV. Despite the fact that iodine was not observed, the degradation of the SAMs on the two different substrates was successfully compared, and as expected, the SAM degrades more on the TiO_2_ substrate due to its superior photocatalytic nature compared to SiO_2_.

For the SiO_2_ substrate, the degradation of the SAM through defluorination is postulated to occur due to OH radical formation. The C-F bonds are cleaved to form CHF-CHF, as reflected by the C 1s spectra. The complete removal of the CF_3_ bonds following one month of ambient exposure is evident, while the F 1s exhibits an obvious difference in the ratio of the two main component peaks after one month. A different degradation mechanism is observed for the SAM on TiO_2_. The excess electrons generated during ambient light exposure disassociate C-F bonds and potentially form C=C bonds, which, in turn, speed up the degradation of the molecule. After one month of ambient exposure, a C=C component peak is visible in the C 1s spectra, verifying the predicted defluorination process. It was observed that the samples of I-PFC12 left in dark conditions with no exposure to ambient light displayed little to no degradation. This confirms that any defluorination is due to exposure to ambient light. Finally, it has been shown that I-PFC12 SAMs are potential candidates for resistless EUV lithography processes as they are easily degraded, even under ambient exposure.

## Figures and Tables

**Table 1:** Atomic concentrations of the I-PFC12 deposited on SiO_2_ when exposed to different durations of ambient and dark conditions.

	C 1s %	O 1s %	F 1s %	Si 2p %
Reference SiO_2_	8.2	35.1	0	56.7
As received	10.6	27.2	19.1	43.1
24 h ambient exp.	10.3	27.9	18.4	43.4
1 week ambient exp.	11.7	27.1	16.7	44.5
1 month ambient exp.	14.2	30.2	12.9	42.7
1 month in dark conditions	14.0	27.5	18.6	39.9
3 months in dark conditions	12.5	29.5	17.3	40.7

**Table 2:** Atomic concentrations of the I-PFC12 deposited on TiO_2_ when exposed to different durations of ambient and dark conditions.

	C 1s %	O 1s %	F 1s %	Ti 2p %
Reference TiO_2_	8.4	67.2	0	24.4
As received	11.5	51.9	17.6	19.0
24 h ambient exp.	11.4	52.3	17.3	19.0
1 week ambient exp.	10.2	53.9	15.2	20.7
1 month ambient exp.	14.4	58.5	7.4	19.7
1 month in dark conditions	11.7	51.9	18.0	18.4
3 months in dark conditions	11.9	50.1	18.3	19.7

## Data Availability

Data sets are available upon reasonable request.

## Supplementary Materials

The following supporting information can be downloaded at: https://www.mdpi.com/article/10.3390/nano14110982/s1, Table S1: Atomic concentrations testing for X-ray damage on both substrates; Figure S1: I 3d, where no evidence of iodine was found on either substrate; Figure S2: (a) O 1s and (b) Si 2p of I-PFC12 on SiO_2_ showing no chemical bonding change after different lengths of ambient exposure; Figure S3: Overlay of the F 1s at each experimental step on SiO_2_; Figure S4: (a) O 1s and (b) Ti 2p of I-PFC12 on TiO_2_ showing no chemical bonding change after different lengths of ambient exposure; Figure S5: An alternative fit of the C 1s including a C=C component peak at 284.4 eV for the I-PFC12 on TiO_2_ after 1 month of ambient light exposure.

## References

[1] Bencher C., Chen Y., Dai H., Montgomery W., Huli L. 22nm Half-Pitch Patterning by CVD Spacer Self Alignment Double Patterning (SADP). Proceedings of the SPIE Advanced Lithography Symposium.

[2] Hobbs R.G., Petkov N., Holmes J.D. (2012). Semiconductor Nanowire Fabrication by Bottom-up and Top-down Paradigms. Chem. Mater..

[3] Vandeweyer T., Bekaert J., Ercken M., Gronheid R., Miller A., Truffert V., Verhaegen S., Versluijs J., Wiaux V., Wong P. Immersion Lithography and Double Patterning in Advanced Microelectronics. Proceedings of the International Conference on Micro- and Nano-Electronics.

[4] Moore G.E. (1998). Cramming More Components onto Integrated Circuits. Proc. IEEE.

[5] Schaller R.R. (1997). Moore’s Law: Past, Present, and Future. IEEE Spectr..

[6] Sharma E., Rathi R., Misharwal J., Sinhmar B., Kumari S., Dalal J., Kumar A. (2022). Evolution in Lithography Techniques: Microlithography to Nanolithography. Nanomaterials.

[7] Mackus A.J.M., Merkx M.J.M., Kessels W.M.M. (2019). From the Bottom-Up: Toward Area-Selective Atomic Layer Deposition with High Selectivity. Chem. Mater..

[8] Gabor A.H., Felix N.M. (2019). Overlay Error Statistics for Multiple-Exposure Patterning. J. Micro/Nanolithogr. MEMS MOEMS.

[9] Peterhänsel S., Gödecke M.L., Paz V.F., Frenner K., Osten W. (2015). Detection of Overlay Error in Double Patterning Gratings Using Phase-Structured Illumination. Opt. Express.

[10] Bhattacharyya K. Tough Road Ahead for Device Overlay and Edge Placement Error. Proceedings of the SPIE Advanced Lithography Symposium.

[11] Harriott L.R. (2001). Limits of Lithography. Proc. IEEE.

[12] Iyengar V.V., Chandrasekaran S., Weddington D., Nettles M.M., Eagle O.H., Tey S.H., Parry T.B. Collapse-Free Patterning of High Aspect Ratio Silicon Structures for 20nm NAND Flash Technology. Proceedings of the 2015 26th Annual SEMI Advanced Semiconductor Manufacturing Conference (ASMC).

[13] Jeong K., Kahng A.B., Topaloglu R.O. Assessing Chip-Level Impact of Double Patterning Lithography. Proceedings of the 2010 11th International Symposium on Quality Electronic Design (ISQED).

[14] Bakshi V. (2009). EUV Lithography.

[15] Levinson H.J. (2022). High-NA EUV Lithography: Current Status and Outlook for the Future. Jpn. J. Appl. Phys..

[16] Manouras T., Argitis P. (2020). High Sensitivity Resists for EUV Lithography: A Review of Material Design Strategies and Performance Results. Nanomaterials.

[17] Closser K.D., Ogletree D.F., Naulleau P., Prendergast D. (2017). The Importance of Inner-Shell Electronic Structure for Enhancing the EUV Absorption of Photoresist Materials. J. Chem. Phys..

[18] Kostko O., Xu B., Ahmed M., Slaughter D.S., Frank Ogletree D., Closser K.D., Prendergast D.G., Naulleau P., Olynick D.L., Ashby P.D. (2018). Fundamental Understanding of Chemical Processes in Extreme Ultraviolet Resist Materials. J. Chem. Phys..

[19] Pasquali M., Brady-Boyd A., Leśniewska A., Carolan P., Conard T., O’Connor R., De Gendt S., Armini S. (2023). Area-Selective Deposition of AlOx and Al-Silicate for Fully Self-Aligned via Integration. ACS Appl. Mater. Interfaces.

[20] Parsons G.N., Clark R.D. (2020). Area-Selective Deposition: Fundamentals, Applications, and Future Outlook. Chem. Mater..

[21] Yarbrough J., Shearer A.B., Bent S.F. (2023). Molecule Inhibitors for Area-Selective Atomic Layer. J. Vac. Sci. Technol. A.

[22] Johnson R.W., Hultqvist A., Bent S.F. (2014). A Brief Review of Atomic Layer Deposition: From Fundamentals to Applications. Mater. Today.

[23] Bobb-Semple D., Nardi K.L., Draeger N., Hausmann D.M., Bent S.F. (2019). Area-Selective Atomic Layer Deposition Assisted by Self-Assembled Monolayers: A Comparison of Cu, Co, W, and Ru. Chem. Mater..

[24] Schreiber F. (2000). Structure and Growth of Self-Assembling Monolayers. Prog. Surf. Sci..

[25] Bain C.D., Whitesides G.M. (1989). A Study by Contact Angle of the Acid-Base Behavior of Monolayers Containing ω-Mercaptocarboxylic Acids Adsorbed on Gold: An Example of Reactive Spreading. Langmuir.

[26] Bain C.D., Troughton E.B., Tao Y.T., Evall J., Whitesides G.M., Nuzzo R.G. (1989). Formation of Monolayer Films by the Spontaneous Assembly of Organic Thiols from Solution onto Gold. J. Am. Chem. Soc..

[27] Nuzzo R.G., Allara D.L. (1983). Adsorption of Bifunctional Orgnaic Disulfides on Gold Surfaces. J. Am. Chem. Soc..

[28] Jo K., Yang H. (2014). Comparative Study of Stability of Phosphonate Self-Assembled Monolayers on Indium-Tin Oxide Electrodes Prepared Using Different Methods. J. Electroanal. Chem..

[29] Jennings G.K., Laibinis P.E. (1996). Self-Assembled Monolayers of Alkanethiols on Copper Provide Corrosion Resistance in Aqueous Environments. Colloids Surf. A Physicochem. Eng. Asp..

[30] Lee S., Shon Y.-S., Colorado R., Guenard R.L., Lee T.R., Perry S.S. (2000). The Influence of Packing Densities and Surface Order on the Frictional Properties of Alkanethiol Self-Assembled Monolayers (SAMs) on Gold: A Comparison of SAMs Derived from Normal and Spiroalkanedithiols. Langmuir.

[31] de Boer B., Hadipour A., Mandoc Magdalena M., van Woudenbergh T., Blom P. (2005). Tuning of Metal Work Functions with Self-Assembled Monolayers. Adv. Mater..

[32] Yan L., Zhao X.M., Whitesides G.M. (1998). Patterning a Preformed, Reactive SAM Using Microcontact Printing. J. Am. Chem. Soc..

[33] Ghezzi M., Thickett S.C., Neto C. (2012). Early and Intermediate Stages of Guided Dewetting in Polystyrene Thin Films. Langmuir.

[34] Zhao B., Moore J.S., Beebe D.J. (2001). Surface-Directed Liquid Flow inside Microchannels. Science.

[35] Gau H., Herminghaus S., Lenz P., Lipowsky R. (1999). Liquid Morphologies on Structured Surfaces: From Microchannels to Microchips. Science.

[36] Mrksich M., Chen C.S., Xia Y., Dike L.E., Ingber D.E., Whitesides G.M. (1996). Controlling Cell Attachment on Contoured Surfaces with Self-Assembled Monolayers of Alkanethiolates on Gold. Proc. Natl. Acad. Sci. USA.

[37] Lodha J.K., Pollentier I., Conard T., Vallat R., De Gendt S., Armini S. (2022). Self-Assembled Monolayers as Inhibitors for Area-Selective Deposition: A Novel Approach towards Resist-Less EUV Lithography. Appl. Surf. Sci..

[38] Shou K., Hong J.K., Wood E.S., Hook J.M., Nelson A., Yin Y., Andersson G.G., Abate A., Steiner U., Neto C. (2019). Ultralow Surface Energy Self-Assembled Monolayers of Iodo-Perfluorinated Alkanes on Silica Driven by Halogen Bonding. Nanoscale.

[39] Abate A., Dehmel R., Sepe A., Nguyen N.L., Roose B., Marzari N., Hong J.K., Hook J.M., Steiner U., Neto C. (2019). Halogen-Bond Driven Self-Assembly of Perfluorocarbon Monolayers on Silicon Nitride. J. Mater. Chem. A.

[40] Cavallo G., Metrangolo P., Milani R., Pilati T., Priimagi A., Resnati G., Terraneo G. (2016). The Halogen Bond. Chem. Rev..

[41] Gutzler R., Fu C., Dadvand A., Hua Y., MacLeod J.M., Rosei F., Perepichka D.F. (2012). Halogen Bonds in 2D Supramolecular Self-Assembly of Organic Semiconductors. Nanoscale.

[42] Wang F., Ma N., Chen Q., Wang W., Wang L. (2007). Halogen Bonding as a New Driving Force for Layer-by-Layer Assembly. Langmuir.

[43] Desiraju G.R., Ho P.S., Kloo L., Legon A.C., Marquardt R., Metrangolo P., Politzer P., Resnati G., Rissanen K. (2023). Definition of the Halogen Bond. Chem. Int..

[44] Ochiai T., Iizuka Y., Nakata K., Murakami T., Tryk D.A., Koide Y., Morito Y., Fujishima A. (2011). Efficient Decomposition of Perfluorocarboxylic Acids in Aqueous Suspensions of a TiO_2_ Photocatalyst with Medium-Pressure Ultraviolet Lamp Irradiation under Atmospheric Pressure. Ind. Eng. Chem. Res..

[45] Wang Y., Wang C., Luo P., Hu Q. (2023). Removal of Perfluorooctanoic Acid by MWCNT-Modified Carbon-Doped Titanium Dioxide in a Peroxymonosulfate/Simulated Sunlight System. Appl. Surf. Sci..

[46] Shirley D.A. (1972). High-Resolution X-Ray Photoemission Spectrum of the Valence Bands of Gold. Phys. Rev. B.

[47] Brady-Boyd A., O’Connor R., Armini S., Selvaraju V., Pasquali M., Hughes G., Bogan J. (2022). The Role of Atomic Oxygen in the Decomposition of Self-Assembled Monolayers during Area-Selective Atomic Layer Deposition. Appl. Surf. Sci..

[48] Satulu V., Ionita M.D., Vizireanu S., Mitu B., Dinescu G. (2016). Plasma Processing with Fluorine Chemistry for Modification of Surfaces Wettability. Molecules.

[49] Lee J., Woo H., Kwon K. (2021). Applied Surface Science Sidewall Chemical Analysis of Plasma-Etched Nano-Patterns Using Tilted X-Ray Photoelectron Spectroscopy Combined with in-Situ Ion Sputtering. Appl. Surf. Sci..

[50] Yang H., Guo J., Sathe C., Agui A., Nordgren J. (1998). Structural and Electronic Properties of Low Dielectric Constant Fluorinated Amorphous Carbon Films. Appl. Phys. Lett..

[51] Tressaud A., Durand E., Labrugère C., Alexander P., Simbirtseva G.V., Kharitonova L.N., Dubois M., Tressaud A., Durand E., Labrugère C. (2013). Surface Modification of Polymers Treated by Various Fluorinating Media. Acta Chim. Slov..

[52] Chen G., Zhang J., Yang S. (2008). Fabrication of Hydrophobic Fluorinated Amorphous Carbon Thin Films by an Electrochemical Route. Electrochem. Commun..

[53] Schneider J., Matsuoka M., Takeuchi M., Zhang J., Horiuchi Y., Anpo M., Bahnemann D.W. (2014). Understanding TiO_2_ Photocatalysis Mechanisms and Materials. Chem. Rev..

[54] Yamijala S.S.R.K.C., Shinde R., Wong B.M. (2020). Real-Time Degradation Dynamics of Hydrated per- And Polyfluoroalkyl Substances (PFASs) in the Presence of Excess Electrons. Phys. Chem. Chem. Phys..

[55] Vinoda B.M., Vinuth M., Bodke Y.D., Manjanna J. (2015). Photocatalytic Degradation of Toxic Methyl Red Dye Using Silica Nanoparticles Synthesized from Rice Husk Ash. J. Environ. Anal. Toxicol..

[56] Liu J., Van Hoomissen D.J., Liu T., Maizel A., Huo X., Fernández S.R., Ren C., Xiao X., Fang Y., Schaefer C.E. (2018). Reductive Defluorination of Branched Per- and Polyfluoroalkyl Substances with Cobalt Complex Catalysts. Environ. Sci. Technol. Lett..

