# Correction: Colbeck Kirby et al. Degradation of Perfluorododecyl-Iodide Self-Assembled Monolayers upon Exposure to Ambient Light. *Nanomaterials* 2024, *14*, 982

**DOI:** 10.3390/nano14181512

**Published:** 2024-09-18

**Authors:** Lauren Colbeck Kirby, Jayant K. Lodha, Simon Astley, Dave Skelton, Silvia Armini, Andrew Evans, Anita Brady-Boyd

**Affiliations:** 1 Physics Department, Aberystwyth University, Aberystwyth SY23 3BZ, UK; 2 Semiconductor Technology and Systems, IMEC, Kapeldreef 75, B-3001 Leuven, Belgium

## Error in Figure

In the original publication [1], there was a mistake in Figure 5 as published. In the last editing step, an incorrect figure was input for Figure 5 (Figure 3 is copied twice) and was not noticed in the final proofread. The corrected Figure 5 appears below. The authors state that the scientific conclusions are unaffected. This correction was approved by the Academic Editor. The original publication has also been updated.


