# Supplementary material for: Degradation of Perfluorododecyl-Iodide Self-Assembled Monolayers upon Exposure to Ambient Light
Source: Nanomaterials (Basel). 2024 Jun 5;14(11):982. doi: 10.3390/nano14110982 (PMC11173715; doi:10.3390/nano14110982)
Supplement: Supplementary file 1 [file nanomaterials-14-00982-s001.zip › Supplementary Information.pdf]

# Degradation of Perfluorododecyl-Iodide Self-Assembled Monolayers upon Exposure to Ambient Light

**Lauren Colbeck Kirby [1], Jayant K. Lodha [2], Simon Astley [1], Dave Skelton [1], Silvia Armini [2], Andrew Evans [1] and Anita Brady-Boyd [1,*]**

[1] Physics Department, Aberystwyth University, Aberystwyth SY23 3BZ, UK
[2] Semiconductor Technology and Systems, IMEC, Kapeldreef 75, B-3001 Leuven, Belgium
* Correspondence: anb116@aber.ac.uk

The follow Table S1, displays the atomic concentrations following prolonged exposure to the x-ray source. After 2 hours of X-ray exposure there is little evidence of damage observed on the $SiO_2$ substrate. A small decrease in the F 1s is observed on the $TiO_2$ substrate.

Table S1: Atomic concentrations testing for X-ray damage on both substrates.

| **$SiO_2$** | **C 1s %** | **O 1s %** | **F 1s %** | **Si 2p %** | **I 3d %** |
|---|---|---|---|---|---|
| **Initial Survey** | 10.9 | 28.6 | 18.9 | 41.6 | 0 |
| **Survey Scan 5** | 9.6 | 29.3 | 16.5 | 44.6 | 0 |
| **Final Survey** | 9.7 | 31.1 | 16.2 | 43.0 | 0 |
| **$TiO_2$** | **C 1s %** | **O 1s %** | **F 1s %** | **Ti 2p %** | **I 3d %** |
| **Initial Survey** | 12.5 | 50.9 | 18.5 | 18.1 | 0 |
| **Final Survey** | 10.5 | 53.0 | 16.5 | 20.0 | 0 |

Figure S1 below shows the absence of the iodine head group of the SAM. No iodine was observed for either substrate at any point during the experiment.

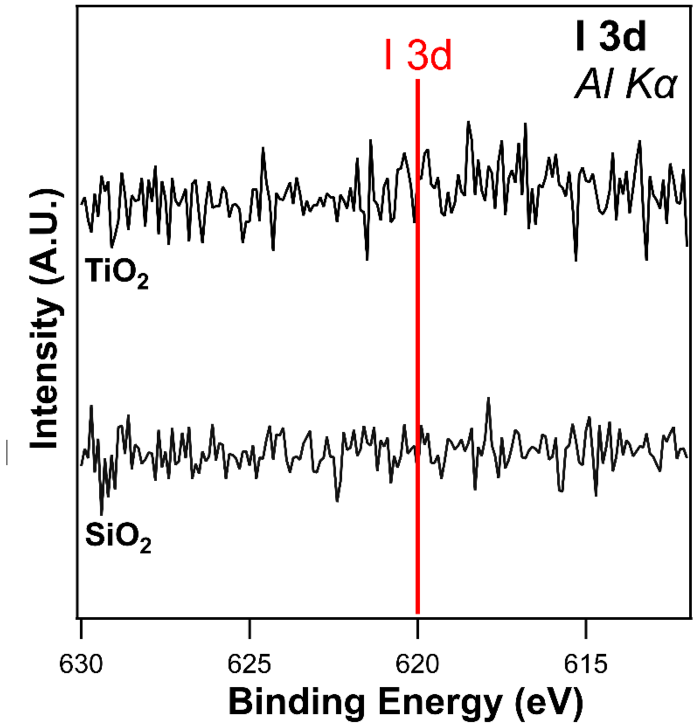


Figure S1: I 3d where no evidence of iodine was found on either substrate.

On the $SiO_2$ substrate no change is observed in either the Si 2p or O 1s, Figure S3.

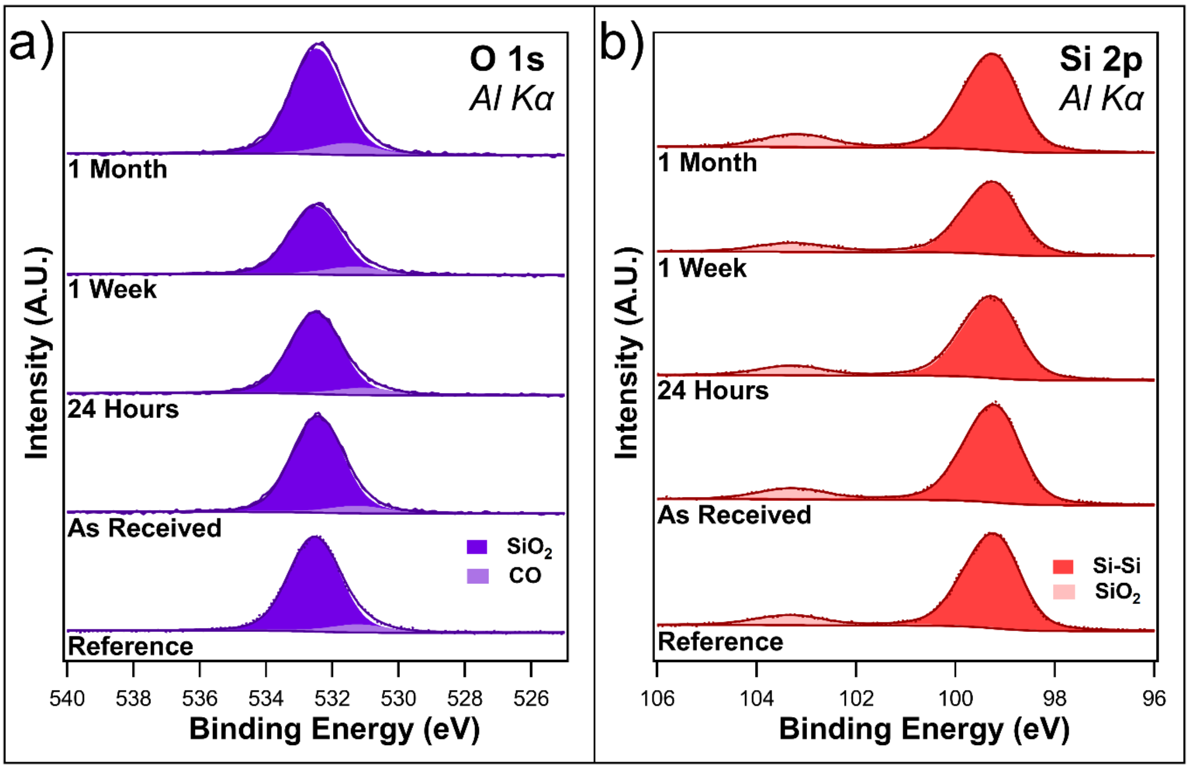


Figure S2: (a) O 1s and (b) Si 2p of I-PFC12 on $SiO_2$ showing no chemical bonding change different lengths of ambient exposure.

Figure S3 below shows the overlay of the unnormalized F1s spectra of the I-PFC12 on $SiO_2$. A consistent decrease in the intensity of the peak is clear with each longer exposure to ambient conditions.

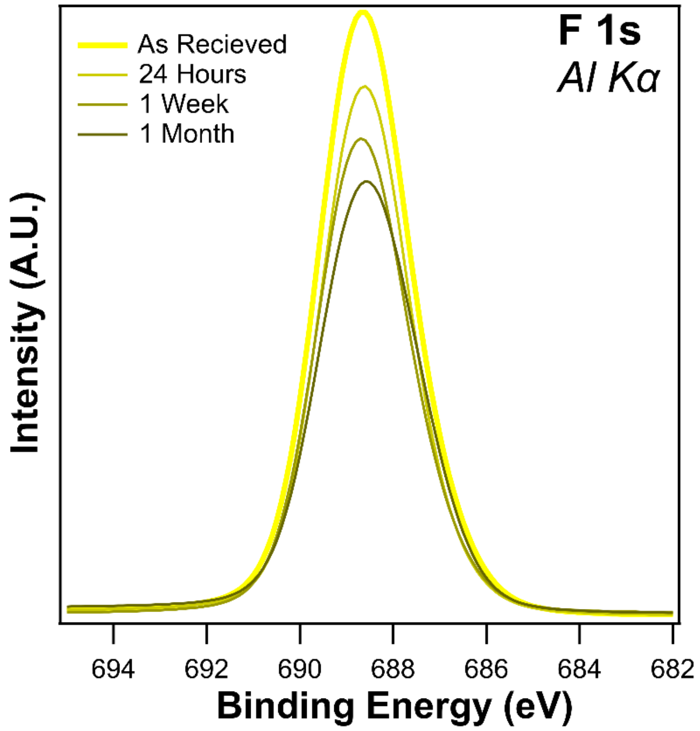


Figure S3: Overlay of the F1s at each experimental step on $SiO_2$.

No change in O 1s or Ti 2p is detected as shown in Figure S4. In the O 1s both component peaks are attributed to titanium dioxide. Figure S4b shows a text book $TiO_2$ Ti 2p spectra.

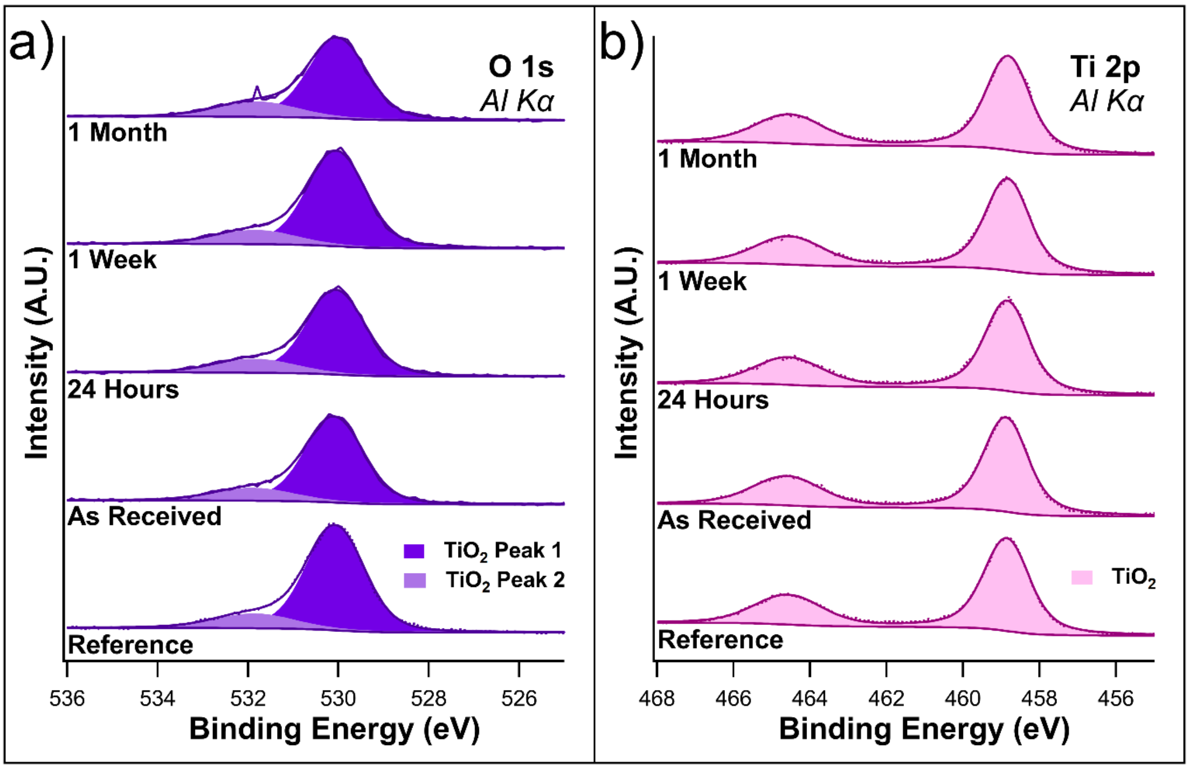


Figure S4: (a) O 1s and (b) Ti 2p of I-PFC12 on TiO2 showing no chemical bonding change after different lengths of ambient exposure.

Following one month exposure of the I-PFC12 on $TiO_2$ there is the potential to insert a C=C component peak on the lower binding energy side of main C-C/C-H component peak. Due to the poor signal to noise we have decided to remain conservative on our approach tp peak fitting.

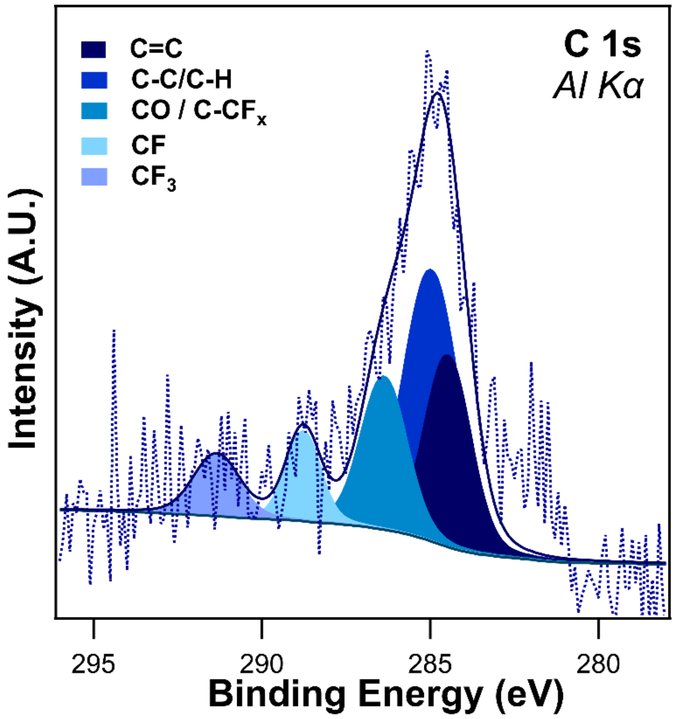


Figure S5: An alternative fit of the C1s including a C=C component peak at 284.4 eV for the I-PFC12 on $TiO_2$ after 1 month of ambient light exposure.
